# Translation and cultural adaptation to Portuguese of Food Allergy Quality of Life Questionnaire-Parent Form (FAQLQ-PF)

**DOI:** 10.1186/2045-7022-5-S3-P101

**Published:** 2015-03-30

**Authors:** Mariana Couto, Diana Silva, Susana Piedade, Audrey Dunn Galvin, Bertine Flokstra - de Blok, Luís Miguel Borrego, Mário Morais-Almeida

**Affiliations:** 1 Instituto CUF and Hospital CUF Porto, Porto, Portugal; 2 Centro Hospitalar São João EPE, Porto, Portugal; 3 Hospital CUF Descobertas, Lisbon, Portugal; 4 University College, Cork, Ireland; 5 University Medical Center Groningen, Groningen, The Netherlands

## Background

Food allergy is a health problem with significant negative impact in Quality of Life (QoL). Currently, there is a growing interest in health assessment tools produced and validated throughout the world and able to quantify this subjective outcome. Nevertheless, food allergy mostly affects children who are not able to report QoL impact, and parents are usually the intermediates.

## Aim

To translate into Portuguese and culturally adapt to our population the Food Allergy Quality of Life Questionnaire - Parent Form (FAQLQ-PF) (0-12years).

## Methods

Cross-cultural translation was performed according to guidelines (Allergy 2010;65:290-295). Linguistic validation consisted in 3 steps: forward translation, backward translation and comprehensibility testing. Two independent translations were performed and a combined consensual version was obtained with an expert support. Subsequently, back-translation was performed and sent to the original author of FAQLQ-PF. After feedback, a consensual version was obtained and tested in parents of food allergic children by cognitive debriefing.

## Results

12 questionnaires were fulfilled, 11 by mothers and 1 by a father. All took 15 min to complete it. No comments, doubts or suggestions were done, except for 2 parents regarding a question about the number of food their children have to avoid. Two gave special positive feedback about the suitability and utility of FAQLQ-PF. Parents of 3 children (all aged <3y) considered that parts of FAQLQ-PF were not adequate to their children given their age.

Among children, 7 were aged <4y, 2 were aged 4-7y, and 3 aged >7y. All had documented food allergy. Clinical symptoms included anaphylaxis (n=9) or only mucocutaneous involvement (n=3). Implicated foods were cow/s milk (n=7), egg (n=7), tree nuts (n=1), fish (n=1) and shrimp (n=1); 5 children had more than one food allergies.

## Conclusion

The Portuguese version of the FAQLQ-PF has been adapted for use in the Portuguese population. Changes have been included after this pre-test in accordance to doubts and suggestions of participants; it can be used after further analysis of their clinimetric properties, which is underway.

